# Supplementary material for: Content-rich biological network constructed by mining PubMed abstracts
Source: BMC Bioinformatics. 2004 Oct 8;5:147. doi: 10.1186/1471-2105-5-147 (PMC528731; doi:10.1186/1471-2105-5-147)
Supplement: Additional File 2 — The original results of the above study (non-essential files are deleted to keep the file size under the limit set by BMC bioinformatics). [file 1471-2105-5-147-S2.bz2 › chilibotAdditionalFile2/dip20/246ID8962075E809/html/RECO_RECF.html]

 


 **RECO** and **RECF** 
  
Found 63 abstracts in PubMed, retrieved 20.  
 

 What does Google say? 
 PDF only 
| .edu only 

---

**Interactive relationship** (e.g. stimulation, inhibition, etc)

**Neutral relationship**- **RecF**  protein competes with  **RecO**  protein for RecR protein association and its addition destabilizes RecAOR filaments.  Ref: 11743007 EMBO J, 2001
- We therefore assigned  **recO**  to epistatic group alpha and predict that the  **RecO**  protein acts at the same stage of recombination as the  **RecF** , RecL and RecR proteins, in a RecFLOR complex.  Ref: 10323239 Mol Gen Genet, 1999

**Non-interactive relationship** (e.g. studied together, co-existance, homology, etc.)

- The  **recO**  mutation did not affect intermolecular recombination in  **recF** , recL, recH or recU cells, but reduced by about 9 fold the incidence of intermolecular recombination in addAB cells.  Ref: 10323239 Mol Gen Genet, 1999
- This action of RecA does not involve the induction of the SOS response and does not require any of the recombination proteins essential for the presynaptic step of homologous recombination, RecBCD,  **RecF**  or  **RecO** .  Ref: 11069680 Mol Microbiol, 2000
- The  **recO**  mutation did not affect intramolecular recombination in the addAB, recU,  **recF**  or recL cells, but reduced it by about 75 fold in recH cells.  Ref: 10323239 Mol Gen Genet, 1999
- In Escherichia coli, the recovery of replication following UV induced DNA damage is dependent upon several proteins in the  **recF**  pathway, including  **RecF** ,  **RecO** , and RecR.  Ref: 10589843 Mol Gen Genet, 1999
- Expression of a gene s from the nin region of phage lambda partially complemented both the viability and recombination defects of the  **recF** ,  **recO** , and recR mutants and the recombination defect of ruvC but not of ruvAB or recQ mutants.  Ref: 10735883 J Bacteriol, 2000
- ii this pathway s requires functional recA,  **recF** ,  **recO** , and recR genes, suggesting the mechanism to be daughter strand gap repair.  Ref: 11073901 J Bacteriol, 2000
- Gap repair genes  **recF** ,  **recO** , and recR were not required.  Ref: 10224240 Genetics, 1999
- The protective effect of plasmid pGam18 against radiation was completely abolished by mutations in the most  **RecF**  pathway genes  **recF** , recJ, recR,  **recO** , recQ, recN, and ruvB.  Ref: 10368783 Genetika, 1999
- The defects in restart attributed to mutations in  **recF** ,  **recO**  or recR were suppressed in a recA730 lexA Def strain expressing constitutively activated RecA RecA\*.  Ref: 11929519 Mol Microbiol, 2002
- The results of these studies suggest that recombinational repair genes, particularly  **recF** , sbcB and  **recO**  have different roles in the induced excision of Tn10 than in recombinational mating.  Ref: 10102036 Mutat Res, 1999
- Finally, examples are shown for in vitro reactions involving DNA, RecA,  **RecO** ,  **RecF** , RecR, and SSB that lead to a further understanding of recombinational repair.  Ref: 11098470 Mol Biotechnol, 2000
- The  **recO**  mutation did not affect the sensitivity of  **recF** , recR or recL cells to DNA damaging agents, increased the sensitivity of recU and addAB cells and abolished the DNA repair capacity of recH cells.  Ref: 10323239 Mol Gen Genet, 1999
- Expression of the Escherichia coli mutA mutator phenotype requires recA, recB, recC, ruvA, and ruvC gene, but not recD,  **recF** ,  **recO** , or recR genes.  Ref: 10671469 J Bacteriol, 2000
- In this study, we find that other genes belonging to the RecBC and  **RecF**  sexual recombination pathways also participate in this process since mutations in recB, sbcB or  **recO**  diminish, though to different degrees, the frequency of Tn10 precise excision induced by MMC treatment or by uvrD mutants.  Ref: 10102036 Mutat Res, 1999
- Resistance of E. coli strains to cisplatin as determined by plating experiments decreased in the following order parent PQ30 strain, polB >  **recO** , recR,  **recF**  > recB.  Ref: 12531023 DNA Repair Amst , 2002
- The Escherichia coli  **RecF** ,  **RecO**  and RecR pro teins have previously been implicated in bacterial recombinational DNA repair at DNA gaps.  Ref: 11743007 EMBO J, 2001
- ii In a recBC sbcA background, in which RecE mediated recombination is active, recA, recJ, recQ, recE, recT,  **recF** ,  **recO** , and recR mutations led to their accumulation.  Ref: 12718760 BMC Mol Biol< MedlineTA>BMC Mol Biol, 2001
- Genetic evidence suggests that the  **RecF** ,  **RecO** , and RecR RecFOR proteins participate in a common step of DNA recombination and repair, yet the biochemical event requiring collaboration of all three proteins is unknown.  Ref: 12769856 Mol Cell, 2003
- We compared the recombination proficiencies of recB1080,  **recO** , recR, and  **recF**  single mutants and recB1080  **recO** , recB1080 recR, and recB1080  **recF**  double mutants.  Ref: 12618388 Genetics, 2003
- Similarly,  **recF** ,  **recO**  or recR mutations suppress the lethality of the Escherichia coli rep uvrD double mutant.  Ref: 12065426 EMBO J, 2002
- dnaC suppressors of priA overcome this inviability, especially when  **RecF** ,  **RecO**  or RecR is inactivated, indicating that RdgC avoids or counters a toxic effect of these proteins.  Ref: 12554673 EMBO J, 2003
- Although the MRF alpha 2 components are apparently not encoded by currently known homologous recombination genes such as recA,  **recF** ,  **recO** , and recR, they may fulfill an important function in assembling replisomes on arrested replication forks and products of homologous strand exchange.  Ref: 11459960 Proc Natl Acad Sci U S A, 2001
- To elucidate the role of several key proteins involved in this process, we have analysed the time dependence of replication restart in strains carrying a combination of mutations in lexA, recA, polB pol II, umuDC pol V, priA, dnaC,  **recF** ,  **recO**  or recR.  Ref: 11929519 Mol Microbiol, 2002
- In a wild type, lexA background, mutations in  **recF** ,  **recO**  or recR block both pathways.  Ref: 11929519 Mol Microbiol, 2002
- previously isolated mutations in B.subtilis  **recF** , recL,  **recO**  and recR, which belong to the same complementation group, all suppress the lethality of a pcrA mutation.  Ref: 12065426 EMBO J, 2002
- This function is countered by the  **RecF**   **RecO**  competition for association with the RecR protein.  Ref: 11743007 EMBO J, 2001
- In this genetic background, mutations in  **recF** ,  **recO** , and recR had large effects on both cell viability and on recombination.  Ref: 10735883 J Bacteriol, 2000
